# Validation of the Patient Health Questionnaire-9 for Screening Depressive Disorders among Korean Employees: A Longitudinal Study of the National Health Examination Data

**DOI:** 10.3390/ijerph19073780

**Published:** 2022-03-22

**Authors:** Jihye Lee, Kyeong-Eun Lee, Sungkyun Park, Kyo Yeon Jun

**Affiliations:** Occupational Safety and Health Research Institute, Korea Occupational Safety and Health Agency, Ulsan 44429, Korea; kyeong85@kosha.or.kr (K.-E.L.); psk@kosha.or.kr (S.P.); reinj@kosha.or.kr (K.Y.J.)

**Keywords:** depression, national health examination, patient health questionnaire-9, screening

## Abstract

This nationwide longitudinal study examined the screening utility of the Patient Health Questionnaire-9 (PHQ-9) for Korean workers (aged 20, 30, 40, 50, 60, and 70 years) who completed the questionnaire in 2018. Data on disease names and health-related behaviors were collected from the National Health Insurance Service (NHIS). Follow-up began on 1 January 2018, and the primary endpoint was the hospitalization date for depression, self-harm, or suicide or 31 December 2019. Of the 766,351 participants, 741,423 received depression screening. Those screened were classified into normal (n = 716,760) and high-risk groups (n = 24,663) based on PHQ-9 scores. The incidence of hospital admissions for depression, self-harm, or suicide in the non-screened, normal, and high-risk groups was analyzed, and the PHQ-9′s validity was examined. There were more females in the high-risk group than in the normal group, and the income distribution differed. The two-year cumulative incidence was highest for the high-risk group (4.21%), followed by the normal (0.89%) and non-screened groups (0.80%). The PHQ-9′s sensitivity was low (males: 14.2%; females: 13.8%). Its specificity for males and females was 97.1% and 96.3%, respectively. Our findings may help develop a system to prevent suicides and hospitalizations attributed to workplace depression.

## 1. Introduction

Korea’s rapid shift from a manufacturing-oriented industrial society to a service-based one that requires restraint of emotions and expression of specific emotions has increased workers’ emotional strain. At the same time, there is increasing emphasis on the need for mental health management of workers owing to the increasing prevalence of mental stress and health problems. However, the occupational risk factors of psychiatric disorders are not clear, and quantification of these factors can be challenging. In addition, because responses to external stress vary with social and individual factors, it is difficult to apply the existing workers’ health management system, which focuses on controlling exposure to hazardous substances.

Several studies have reported that job stress damages mental health and increases the risk for depression and suicide [1,2,3,4,5]. A Japanese study evaluated a worker support program and reported a reduction of suicide risk in 86% of workers with pre-existing suicidal thoughts; their findings indicated the need for active interventions to manage workers’ mental health problems [6].

However, few studies have assessed screening tests and follow-up management methods to effectively manage psychiatric disorders among workers. In particular, evidence on industries vulnerable to psychiatric disorders and work-related risk factors is lacking, making it difficult to actively evaluate and manage mental health within the workplace. According to a study conducted in Japan, lack of control and poor human relations at workplaces are associated with persistent depressive symptoms among blue-collar workers [5]. Most studies of occupational health effects conducted in Korea to date have focused on the effects of tangible exposure to hazardous substances within the manufacturing industry. Examples include benzene, formaldehyde, and leukemia [7,8]. Moreover, the definition of work-related stress is diverse, making it difficult to apply the results of previous research on psychiatric disorders. In addition, management of psychiatric disorders is focused on neuroses caused by acute intrinsic factors, such as anxiety disorder, posttraumatic stress disorder, and acute stress disorder, rather than mood disorders, such as depressive disorder.

The National Health Examination is conducted once every two years for office workers and annually for non-office workers in Korea. Although there has been no examination of psychiatric disorders, national health screening programs—including mental health screening—of the 40-year-old population have been conducted since 2007. Individuals with abnormal findings in the primary screening test are classified into a high-risk group for depression. As secondary approaches, the Center for Epidemiologic Studies Depression Scale (CES-D) is administered, and counseling is provided. In 2018, the primary screening instrument was replaced with the Patient Health Questionnaire-9 (PHQ-9), and the participant pool was extended to those aged 40, 50, 60, and 70 years, including those in the National Health Examination database. The PHQ-9 was developed by Spitzer et al. (1999); it is a self-report questionnaire designed to help detect and diagnose psychiatric disorders, such as depression [9]. The PHQ-9 consists of nine questions, which are shorter than the CES-D questions; therefore, it is widely used as a screening tool in clinical practice. The Korean version of the PHQ-9 was standardized by An et al. [10]. However, data are not established because this tool is recent, and its predictive value and sensitivity to identify the actual incidence of psychiatric disorders in workers have not been examined. Therefore, a study on the application of the PHQ-9 since 2018 is necessary.

When developing guidelines for diagnosis, treatment, and prognosis for a specific population, the feasibility—including availability and effectiveness—of existing methods should be reviewed first. If screening tools for mental health management of workers have not been standardized, the preventive effect of screening tools for psychiatric disorders focused on the workers must be reviewed.

We retrospectively investigated the hypothesis that the group at high risk according to the depression screening test (PHQ-9) would have a higher risk of being hospitalized for depression, self-harm, or suicide in the future. The purpose of this study was thus to evaluate the preventive effect of the newly adopted PHQ-9 for Korean workers and to examine its applicability to screen for mental health disorders among workers.

## 2. Methods

### 2.1. Data Source

The National Health Information Database of the National Health Insurance Service (NHIS) includes information on the medical statements, medical histories, and prescription histories of National Health Insurance claimers and medical benefits holders in the Republic of Korea since 2002. Recent data are focused on the start date of treatment. These data consist of qualification, medical treatment, and health examination tables; data are compiled as of 1 January of each year.

### 2.2. Study Population and Materials

The study subjects were those who received depression screening in 2018, when those aged 40, 50, 60, and 70 years were screened for depression. We targeted workers who were born in 1948, 1958, 1968, and 1978 and who were NHIS claimants.

The start date of the follow-up was 1 January 2018. Follow-up ended at hospitalization for depression, self-harm, or suicide or on 31 December 2019. To reduce the effects of psychiatric disorders that already occurred before the time of enrollment, 2016–2017 was set as the wash-out period. As this study focused on severe depression rather than depression managed in outpatient facilities, patients hospitalized for psychiatric disorders during the wash-out period were excluded. Based on previous studies that reported a relationship between the occurrence of depression and the prevalence of other psychiatric disorders, hospitalizations for the Korean Standard Classification of Disease (KCD)-7 codes F20–29 (schizophrenia (schizotypal and delusional disorder)] and F30–39 (mood (affective) disorders) were excluded [11,12,13].

Participants’ data on qualifications, socioeconomic factors, health examinations, and history of hospital and clinic usage in 2018 were collected. To determine whether participants had any diseases at baseline, data on multiple comorbidities corresponding to the Charlson comorbidity index (CCI) were obtained from the medical history table in the 2018 outpatient records [14]. We divided the sample into non-screened and screened groups, and those screened were classified into normal and high-risk groups based on PHQ-9 scores. Persons with a PHQ-9 score of 9 or less were classified into the normal group, whereas those with a score of 10 or more were classified into the high-risk group.

### 2.3. Outcome Measures

In this study, we defined depression as hospitalizations registered for depressive symptoms, self-harm, or suicide. Follow-up was terminated on the date of depression onset. KCD-7 codes F32 (depressive disorder) and F33 (relapsing depressive disorder) were included in the NHIS database. Marshal et al. (2011) reported that mood disorders, such as depression, are prevalent in approximately 60% of suicide deaths, suggesting a strong association between suicide and mood disorders [15]. Therefore, hospitalizations for self-harm or suicide (KCD-7 codes X60–84, intentional self-harm) were included in the depressive disorders category. In the case of repeat hospitalizations, the first day of hospitalization (i.e., the start date of treatment) was considered the date of occurrence. 

At the end of follow-up, the cumulative incidence of depression, self-harm, or suicide was calculated for the non-screened group, the normal group, and the high-risk group. We calculated the sensitivity, specificity, positive predictive value, and negative predictive value of the PHQ-9 for two-year cumulative incidence of depression, self-harm, or suicide.

### 2.4. Statistical Analysis

Since previous studies have reported differences between men and women with psychiatric disorders concerning their behavioral patterns [16,17], the data were stratified by gender in this study. A chi-square test was performed on demographic characteristics, such as gender, income level, and underlying disease status, to compare the differences between the normal and high-risk groups, classified by participants’ scores on the PHQ-9.

A log-rank test was performed to compare the differences in the incidence of depression, self-harm, and suicide between the non-screened, normal, and high-risk groups over time. To determine the extent to which the PHQ-9 predicted the occurrence of depression, self-harm, or suicide during the follow-up period and thereby determine its validity, we assessed the sensitivity, specificity, and positive and negative predictive values of the PHQ-9. 

A *p* < 0.05 indicated statistical significance. Data were analyzed using SPSS (version 25.0; IBM Corp., Armonk, NY, USA) and R version 3.5.1 (R Foundation for Statistical Computing, Vienna, Austria). 

### 2.5. Ethics Declarations

Informed consent was waived because we analyzed secondary data. The study protocol was approved by the Institutional Review Board of the Occupational Safety and Health Research Institute (approval number: OSHRI-202102-HR-001).

## 3. Results

### 3.1. Selection of the Study Participants

**Figure 1:**
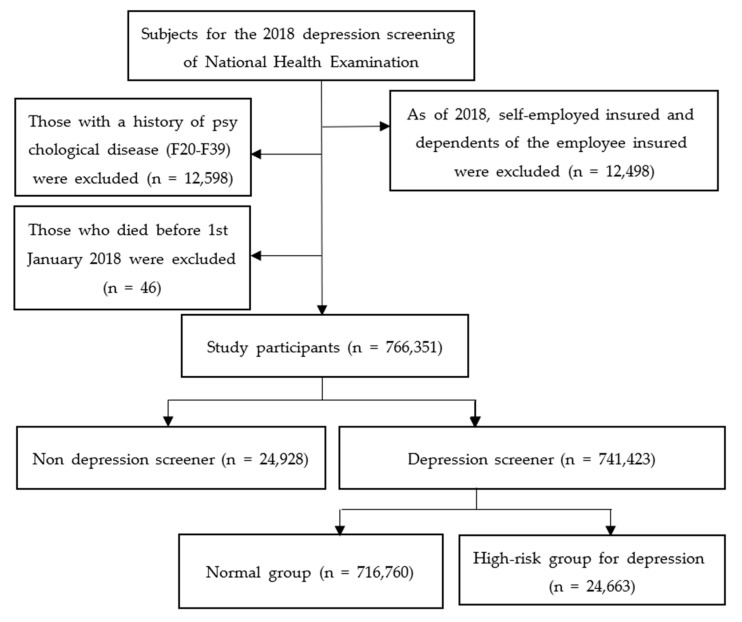
Selection of the study participants.

Figure 1 shows the selection of the study participants. Of the 791,493 people aged 20, 30, 40, 50, 60, and 70 years who received general health examinations in 2018, 12,498 people who were self-employed insured and dependents of the employee-insured were excluded. That is, persons who received general health examinations were included among employer-insured persons who were not self-employed. Further, 12,598 people diagnosed with KCD-7 schizophrenia and mood disorders during the wash-out period were excluded. There was no significant difference in the distribution of the excluded workers between those screened and those not screened for depression (Table 1). Schizophrenia, schizotypal, and delusional disorders were prevalent in 0.09% (22 people) of the non-screened group and 0.12% (924 people) of the screened group. Mood disorders were reported in 1.29% (322 people) of the non-screened group and 1.53% (11,330 people) of the screened group. Of the 766,397 participants, excluding workers with psychiatric disorder, 46 deaths were excluded. Finally, 766,351 workers were included in the analysis, of whom 24,928 people were not screened for depression (non-screen group), and 741,423 workers were screened for depression (screened group).

### 3.2. Demographic Characteristics According to PHQ-9 Scores

Of the 741,423 people who were screened for depression, 716,760 (96.7%) were classified as normal, and 24,663 (3.3%) were classified at high risk according to the PHQ-9. Regarding the gender distribution by group, the proportion of women in the high-risk group was 43.7% (n = 10,783)—significantly higher than the 37.6% (n = 269,454) in the normal group (Table 2).

The distribution of the income quartile based on the National Health Insurance premium was analyzed (Table 3). Among men, the proportion of normal-group participants in the first quartile (i.e., low income) was 13.1% (n = 57,462), which was significantly lower than that in the group at high risk of depression according to PHQ-9 scores (13.3%; n = 1835). Further, the proportion of normal-group participants in the fourth quartile was 41.9% (n = 197,707), which was significantly lower than that in the high-risk group (44.9%; n = 5774). Among women, the proportion of normal-group participants in the first quartile was 37.6% (n = 99,769), which was significantly higher than that in the high-risk group (36.4%; n = 3867 people). Further, the proportion of normal-group participants in the fourth quartile was 18.6% (n = 49,171), which was significantly higher than that in the high-risk group (17.8%; n = 1887).

Regarding chronic diseases divided into subtypes, the distribution of the prevalence rates of some diseases differed between normal and high-risk groups (Table 4). In general, the prevalence of chronic diseases was higher in the high-risk group than in the normal group. Among men in the normal group, the prevalence of peripheral vascular disease was 1.4% (n = 6265), which was significantly higher than that among men in the high-risk group (1.1%; n = 155). In addition, the prevalence rates of gastric ulcer, mild liver disease, and AIDS were 1.8% (n = 7912), 2.7% (n = 12,178), and 0.0% (n = 59), respectively, in the normal group; these were significantly lower than the 2.1% (n = 292), 3.0% (n = 422), and 0.0% (n = 5) in the high-risk group, respectively. Among women in the normal group, the prevalence rates of lower respiratory tract disease and gastric ulcer were 4.5% (n = 12,250) and 2.2% (n = 5916), respectively; these were significantly lower than the 5.3% (n = 576) and 2.6% (n = 284), respectively, among women in the high-risk group.

### 3.3. Comparison of Depression Incidence by Group

During the follow-up period, there were 7634 cases of hospitalization for depression, self-harm, or suicide, and the number of cases in each group is shown in Table 5. Based on the PHQ-9 scores, the two-year cumulative incidence was the highest in the high-risk group (4.21%), followed by the normal (0.89%) and non-screened groups (0.80%). In particular, the cumulative incidence rate in the screened group was 1.00%, which was higher than the 0.80% in the non-screened group. 

The results of the log-rank test to identify any differences in survival rates by group (normal, high-risk, and non-screened) showed that the survival function significantly differed among the three groups (*p* < 0.001). The average survival time and the corresponding log-rank test values for each group are shown in Table 6.

### 3.4. Validation of the PHQ-9

In the depression screening component of the national medical examination, there were significant differences in the cumulative incidence rates of depression, self-harm, and suicide during the follow-up time between the normal, high-risk, and non-screened groups. The sensitivity, specificity, positive predictive value, and negative predictive value of the PHQ-9 for two-year cumulative hospitalizations for depression were calculated; these are presented in Table 7 and Table 8.

Of the total cases, 96.4% of men and 95.2% of women matched the number of cases that coincided with actual number of hospitalizations, and the specificity was high at 97.1% for men and 96.3% for women. However, the sensitivity was low at 14.2% and 13.8% for men and women, respectively.

## 4. Discussion

This study compared the differences between normal and high-risk groups for depression and assessed the validity of the PHQ-9. There were more females in the group at high risk of depression than in the normal group, and there was a difference in the income distribution. This result is consistent with a previous study showing that females are more likely to develop depression during their lifetime than males [18]. 

We used the Korean version of the PHQ-9 as a measurement tool. An et al. (2013) compared the mean score of the PHQ-9 between a group of Koreans with major depressive disorder (MDD) and control group of Koreans [10]. The MDD group had a higher average score (18.57 ± 5.94) than the normal control group (3.19 ± 2.54). Persons with a PHQ-9 score of 9 or less were classified into the normal group, and persons with a score of 10 or more were classified into the high-risk group, consistent with An et al. (2013). The optimal cut-off point was nine with sensitivity 88.5% and specificity 94.7%. This is similar to the sensitivity of 84% and specificity of 92% of the Spanish version of the PHQ-9 [19]. In An et al. (2013), the Cronbach’s alpha measure of internal consistency of the PHQ-9 was high at 0.95, and test-retest reliability was 0.91 [10].

Men with high PHQ-9 scores presented with comorbidities, including peripheral vascular disease, mild liver disease, diabetes with complications, and AIDS. Meanwhile, women at high risk for depression presented with lower respiratory tract disease and gastric ulcer as comorbidities. Zhang et al. reported that hypertension, coronary artery disease, and diabetes are associated with a high incidence of depression, which could affect treatment and prognosis [20]. Depression is a very common risk factor associated with the development and mortality of cardiovascular diseases, especially in older adults [20]. Meanwhile, depression and type 2 diabetes share a common biological origin [21]. In particular, there is growing evidence that depression and diabetes are caused by the over-activation of innate immunity, leading to cytokine-mediated inflammatory responses as well as through dysregulation of the hypothalamic–pituitary–adrenal axis [21].

In this study, depression, self-harm, and suicide were selected as outcome indicators. Depression is the most common psychiatric disorder among people who die by suicide. [22] Boot et al. analyzed 105 treatment-seeking individuals diagnosed with personality disorders who were participating in the Trauma to Personality Spectrum Study (TOPSS) [23]. They additionally analyzed the association between the NEO Five-Factor Inventory and the three-category suicide outcome: non-suicidal, suicidal ideation (SI), and suicide attempts (SA). Of the Big Five traits, the introversion-extraversion dimension most clearly distinguished individuals with SI from non-suicidal individuals as well as those with SA in the past from those with SI only. 

The impact of PHQ-9 on depression, suicide, or self-harm was assessed longitudinally. According to the PHQ-9 scores, the two-year cumulative incidence was the highest in the high-risk group (4.21%), followed by the normal (0.89%) and non-screened groups (0.80%). Andrea et al. (2009) examined the incidence of subclinical anxiety and depression in a general working population [24] in their prospective study that included 3707 workers from the Maastricht Cohort Study on Fatigue at Work. Anxiety and depression were measured using the Hospital Anxiety and Depression Scale. The cumulative 23-month incidence rates of subclinical anxiety and depression were 4.6% and 3.3%, respectively, which was similar to the high-risk depression group in our study. 

Few studies have assessed whether the PHQ-9 is a predictor of severe depression and suicide during follow-up. Simon et al. examined whether responses to the PHQ-9 predicted subsequent suicide attempts or suicide death [25]. After adjustment for age, sex, treatment history, and depression severity, responses to item 9 of the PHQ-9 remained a strong predictor of suicide attempts and suicide death. This excess risk emerged over several days and continued for several months, indicating that suicidal ideation represented an enduring vulnerability. 

Martin-Subero et al. evaluated the impact of depression in medical inpatients on the mortality rate during a 16.5- and 18-year follow-up period [26]. A PHQ-9 score indicating the presence of major depressive disorder predicted increased mortality (hazard ratio (HR), 2.44; 95% CI, 1.39–4.29) after adjusting for demographic and clinical variables. The author suggested that depression severity as represented by the PHQ-9 score is a risk factor for mortality. 

In the present study, the sensitivity of the PHQ-9 was 14.2% for men and 13.8% for women, and the specificity was 97.1% for men and 96.3% for women. Kroenke et al. assessed the validity of the PHQ-9 among 6000 patients across eight primary clinics and seven obstetrics-gynecology clinics [27]. In their study, construct validity was assessed using the 20-item Short-Form General Health Survey, and criterion validity was assessed by a mental health professional (MHP) interview of a sample of 580 patients. In the MHP reinterview, a PHQ-9 score over 10 had a sensitivity of 88% and a specificity of 88% for major depressive disorder. 

The PHQ-9 test used in the depression screening test is a preliminary test to identify at-risk workers for an actual depression diagnostic test; thus, the instrument must possess higher sensitivity than the diagnostic test. Therefore, a test tool with a sensitivity of 13.8–14.2% may not be a suitable screening tool prior to the diagnostic test. However, when individuals are classified as “low risk” based on the PHQ-9, the actual probability of being in the normal group was 98.8–99.3%, which can be evaluated as a meaningful metric. The specificity was very high for both men and women, and the negative predictive value was also high at 99.3% for men and 98.8% for women. In this regard, although it is difficult to screen healthy workers for psychiatric disorders, measures to prevent and manage intentional injuries, such as self-harm and suicide, in the high-risk group for depression may be considered.

This study has some limitations. First, it was conducted using NHIS data, which are secondary, and an operational definition of disease occurrence was used. Although the NHIS data have the advantage of comprising medical examination data of all citizens, these data are generated for administrative purposes rather than for monitoring health issues in the population. Therefore, depending on definitions of diseases, the results may be different. In addition, as this study defined outcome indicators as the occurrence of depression and hospitalization for self-harm or suicide, the sensitivity of the PHQ-9 was likely underestimated. Third, the hospitalization for suicide attempts or self-harm could have been attributed to psychiatric disorders other than depression.

This study evaluated the applicability of using the newly introduced PHQ-9 as a screening tool in the interest of preventing and managing mental health disorders among workers. Studies that analyze various occupational characteristics, such as industry classification, workplace size, and work type, according to the PHQ-9 are needed in the future. These studies can be used as a basis for preventing and managing depression-caused hospitalizations and suicides among workers.

## 5. Conclusions

This study evaluated the usefulness of the PHQ-9, which has been used to screen workers for depression in Korea. During the follow-up period, the incidence of hospitalizations for depression, self-harm, or suicide was the highest in the PHQ-9 high-risk group (4.21%), followed by the normal (0.89%) and non-screened groups (0.80%). The PHQ-9 test was evaluated as inadequate due to its low sensitivity to be used as a tool to screen for risk of hospitalization due to depression, suicide, or self-harm. However, the specificity was very high at 97.1% in males and 96.3% in females, and the negative predictive value was 99.3% in males and 98.8% in females. Even though it would be difficult to use the PHQ-9 as a screening tool to prevent psychiatric diseases in healthy workers, it could be used to prevent and manage intentional injuries, such as suicide and self-harm, in the group at high risk of depression. Our findings may help develop a preventive system for suicides and hospitalizations attributed to workplace depression.

## Figures and Tables

**Table 1:** Prevalence of the psychiatric disorders among workers excluded from the study.

Psychiatric Disorder	Non-Screened		Screened		*p*-Value *
	n	%	n	%	
Schizophrenia, schizotypal, and delusional disorders	22	0.09%	924	0.12%	0.427
Mood disorders	322	1.29%	11,330	1.53%	

* Chi-square test.

**Table 2:** Gender distribution according to the results of the PHQ-9 scores.

		Normal		High Risk for Depression		*p*-Value *
		n	%	n	%	
Gender	Male	447,306	62.4%	13,880	56.3%	<0.001 ***
	Female	269,454	37.6%	10,783	43.7%	

* Chi-square test. *** *p* < 0.001.

**Table 3:** Distribution of income level by gender according to PHQ-9 scores.

Household Income **		Normal		High Risk for Depression		*p*-Value *
		n	%	n	%	
Male	1st quartile	57,462	13.1%	1835	13.3%	<0.001 ***
	2nd quartile	63,397	14.4%	1909	13.9%	
	3rd quartile	120,596	27.5%	4253	30.9%	
	4th quartile	197,707	41.9%	5774	44.9%	
Female	1st quartile	99,769	37.6%	3867	36.4%	<0.001 ***
	2nd quartile	72,066	27.2%	2864	27.0%	
	3rd quartile	44,013	16.6%	1996	18.8%	
	4th quartile	49,171	18.6%	1887	17.8%	

* Chi-square test; ** Calculation of income based on insurance premiums. Bold format *** *p* < 0.001.

**Table 4:** Comparison of the prevalence of comorbidities according to PHQ-9 scores.

Chronic Disease		Normal		High Risk for Depression		*p*-Value *
		n	%	n	%	
Male	Myocardial infarction	703	0.2%	20	0.1%	0.701
	Congenital heart failure	1629	0.4%	43	0.3%	0.294
	Peripheral vascular disease	6265	1.4%	155	1.1%	**0.005**
	Cerebrovascular disease	3012	0.7%	99	0.7%	0.572
	Dementia	313	0.1%	12	0.1%	0.471
	Chronic pulmonary disease	16,447	3.7%	550	4.0%	0.079
	Rheumatic disease	878	0.2%	35	0.3%	0.145
	Peptic ulcer disease	7912	1.8%	292	2.1%	**0.003**
	Mild liver disease	12,178	2.7%	422	3.0%	**0.024**
	Diabetes with chronic complications	5137	1.1%	165	1.2%	0.661
	Hemiplegia or paraplegia	132	0.0%	9	0.1%	0.019
	Renal disease	919	0.2%	30	0.2%	0.784
	Any malignancy (no metastasis), including lymphoma and leukemia, except for malignant neoplasm of skin	2834	0.6%	89	0.6%	0.911
	Moderate or severe liver disease	101	0.0%	3	0.0%	1.000
	Metastatic solid tumor	145	0.0%	6	0.0%	0.488
	AIDS/HIV	59	0.0%	5	0.0%	**0.025**
Female	Myocardial infarction	55	0.0%	2	0.0%	1.000
	Congenital heart failure	568	0.2%	21	0.2%	0.721
	Peripheral vascular disease	3435	1.3%	147	1.4%	0.423
	Cerebrovascular disease	1275	0.5%	51	0.5%	0.997
	Dementia	299	0.1%	15	0.1%	0.392
	Chronic pulmonary disease	12,250	4.5%	576	5.3%	<0.001 ***
	Rheumatic disease	1319	0.5%	61	0.6%	0.268
	Peptic ulcer disease	5916	2.2%	284	2.6%	**0.002**
	Mild liver disease	4139	1.5%	188	1.7%	0.087
	Diabetes with chronic complications	1609	0.6%	55	0.5%	0.249
	Hemiplegia or paraplegia	25	0.0%	1	0.0%	1.000
	Renal disease	236	0.1%	7	0.1%	0.433
	Any malignancy (no metastasis), including lymphoma and leukemia, except malignant neoplasm of skin	2784	1.0%	126	1.2%	0.174
	Moderate or severe liver disease	29	0.0%	0	0.0%	0.628
	Metastatic solid tumor	141	0.1%	5	0.0%	0.790
	AIDS/HIV	0	0.0%	0	0.0%	-

* Chi-square test; Bold format; *** *p*<0.001.

**Table 5:** Cases of hospitalizations for depression, self-harm, or suicide (2018–2019).

Group		n	Number of Cases	Cumulative Incidence Rate at the End of Follow-Up
Screened		741,423	7435	1.00%
	Normal	716,760	6396	0.89%
	High risk for depression	24,663	1039	4.21%
Non-screened		24,928	199	0.80%

**Table 6:** Survival function of the normal, high-risk, and non-screened groups based on the log-rank test.

Group	Average Survival Time (Years)				Log-Rank Test	*p*-Value
	Estimate	Standard Error	95% Confidence Interval			
			Lower	Upper		
Normal	23.883	0.002	23.879	23.886	2703.293	<0.001 ***
High risk for depression	23.528	0.017	23.496	23.561		
Non-screened	23.892	0.009	23.875	23.909		
Total	23.872	0.002	23.868	23.875		

***p < 0.001.

**Table 7:** Depression screening and distribution of cumulative hospitalizations for depression, self-harm, or suicide.

Hospitalization for Depression, Self-Harm, or Suicide		Male		Female	
		Hospitalization (+)	Hospitalization (−)	Hospitalization (+)	Hospitalization (−)
Depression category	Normal	3244 (FN)	444,062 (TN)	3152 (FN)	266,302 (TN)
	High risk for depression	536 (TP)	13,344 (FP)	503 (FP)	10,280 (FP)
Total		3780	457,406	3655	276,582

FN, false negative; TN, true negative; FP, false positive; TN, true negative.

**Table 8:** Validity analysis of the PHQ-9 for predicting hospitalizations for depression, self-harm, or suicide.

Validity	Male		Female	
Sensitivity	14.2%	(536/3780)	13.8%	(503/3655)
Specificity	97.1%	(444,062/457,406)	96.3%	(266,302/276,582)
Positive predictive value	3.9%	(536/13,880)	4.7%	(503/10,783)
Negative predictive value	99.3%	(444,062/447,306)	98.8%	(266,302/269,454)

## Data Availability

The datasets used and/or analyzed during the current study are available from the corresponding author upon reasonable request.
